# A Novel Biodegradable Multilayered Bioengineered Vascular Construct with a Curved Structure and Multi-Branches

**DOI:** 10.3390/mi10040275

**Published:** 2019-04-24

**Authors:** Yuanyuan Liu, Yi Zhang, Weijian Jiang, Yan Peng, Jun Luo, Shaorong Xie, Songyi Zhong, Huayan Pu, Na Liu, Tao Yue

**Affiliations:** 1 School of Mechatronic Engineering and Automation, Shanghai University, Shanghai 200444, China; yuanyuan_liu@shu.edu.cn (Y.L.); zhangyishu@shu.edu.cn (Y.Z.); luojun@shu.edu.cn (J.L.); srxie@shu.edu.cn (S.X.); zhongsongyi@shu.edu.cn (S.Z.); phygood_2001@shu.edu.cn (H.P.); liuna_sia@shu.edu.cn (N.L.); 2 School of Mechanical Engineering, Shanghai Jiao Tong University, Shanghai 200240, China; weijiandjiang@163.com

**Keywords:** bioengineered vascular constructs, curved structure, multi-branches, enzymatically-crosslinked, tissue engineering

## Abstract

Constructing tissue engineered vascular grafts (TEVG) is of great significance for cardiovascular research. However, most of the fabrication techniques are unable to construct TEVG with a bifurcated and curved structure. This paper presents multilayered biodegradable TEVGs with a curved structure and multi-branches. The technique combined 3D printed molds and casting hydrogel and sacrificial material to create vessel-mimicking constructs with customizable structural parameters. Compared with other fabrication methods, the proposed technique can create more native-like 3D geometries. The diameter and wall thickness of the fabricated constructs can be independently controlled, providing a feasible approach for TEVG construction. Enzymatically-crosslinked gelatin was used as the material of the constructs. The mechanical properties and thermostability of the constructs were evaluated. Fluid-structure interaction simulations were conducted to examine the displacement of the construct’s wall when blood flows through it. Human umbilical vein endothelial cells (HUVECs) were seeded on the inner channel of the constructs and cultured for 72 h. The cell morphology was assessed. The results showed that the proposed technique had good application potentials, and will hopefully provide a novel technological approach for constructing integrated vasculature for tissue engineering.

## 1. Introduction

In the field of tissue engineering, one methodology that is often used is to integrate signal factors, cells and biomaterial constructs to replace or improve functional tissues [1,2]. In this common practice, biomaterial constructs act as the basic structure to support and guide cell growth under the influence of some biologically active molecules [3]. An ideal biomimetic structure must have properties (e.g., biocompatibility, elasticity, extensibility) similar to those of the extracellular matrix (ECM) of the native tissue [4]. The selection of the structure and the materials of the biomimetic construct is crucial for effectively mimicking the ECM of human body.

Coronary bifurcation is vulnerable to atherosclerosis as a result of the distinct local blood flow patterns [5] caused by the bifurcated structure of the coronary artery [6]. The blood flow through the artery exerts shear stress onto this particular area, which makes this area susceptible to lesions [7], and which subsequently leads to the coronary artery bifurcation lesion [8], one common lesion of the coronary artery. The treatment of coronary artery bifurcation lesions is a major challenge for interventional cardiology [9]. There exist several interventional techniques for the treatment of this disease, such as the kissing balloon dilatation, crush technique, amongst others [10]. However, the operation process of these techniques remains complex, and the complication rates are usually high. The results of the interventional techniques are usually not satisfactory [11]. For example, the bifurcation area is often subjected to occlusion after operation, and the restenosis rate is very high. Instead of employing traditional interventional techniques, the development of a substitution of the coronary arteries for transplantation is a new method that holds great promise [12].

As far as the treatment of vascular diseases such as the abovementioned coronary bifurcation lesions is concerned, autografts are an ideal candidate in transplantation operations [13]. They possess a benign biocompatibility to the host patients, and the rejection rates are usually low. However, the number of autografts is often rare because of the lack of suitable donors and harvest sites [14]. Synthetic grafts composed of polytetrafluoroethylene and Dacron perform well in replacing large-diameter blood vessels (inner diameter > 6 mm), but they are not suitable for small-diameter conditions [15]. In the channel of small-diameter vessels, the blood flow rate is low and the shear stress is high, which often causes thrombosis and neointimal hyperplasia. In contrast, scaffolds made of natural materials such as gelatin and chitosan often perform better [16].

Some of the recent tissue engineered vascular graft (TEVG) fabricating methods tend to mimic the native blood vessels, which feature a multilayered structure, multi-branches and a spatial configuration [17,18,19]. Like the vessels in vivo, the multilayered structure is helpful for aligning different types of cells, such as endothelial cells, fibroblasts, and smooth muscle cells, in different layers and improving the interaction between them [20], which is a tremendous progress in better mimicking the structure of a native blood vessel. Additionally, in human tissue, the number of vessel branches increases while their inner diameters decrease [21]. This morphology could ensure the mass transportation within the whole vascular network. Therefore, when designing the relatively large-diameter portion of a vessel network, the branching factor must be taken into consideration. Finally, for the purpose of realizing an even distribution of the vessel network in a three-dimensional space, the configuration of the large-diameter channel should be designed as a three-dimensional structure rather than a planar one. However, due to the limitations of the fabrication techniques and materials, it still currently remains a challenge to produce bioengineered vascular constructs that meet all of the abovementioned characteristics.

Among materials commonly used for biological tissue fabrication, gelatin is a natural polymer similar to collagen; it possesses benign biocompatibility and biodegradability [22,23]. Due to these properties, gelatin could be an ideal candidate for cell delivery and could serve as the substitute for ECM. Some recent studies have used microbial transglutaminase (mTG) to crosslink gelatin to increase the biocompatibility and mechanical strength of engineered constructs [24,25,26]. Enzymatically crosslinked gelatin gels were proven to be biocompatible with human cells. For this reason, a gelatin/mTG biopolymer was usually chosen as the ECM-like material for fabricating biomimetic constructs. Besides, Pluronic F127, which is composed of polyoxyethylene (PEO) and polyoxypropylene (PPO), undergoes thermally reversible gelation above a lower critical solution temperature (LCST) [27,28]. Therefore, it is suitable as the fugitive ink when constructing a hollow channel.

In the present study, a novel additive and subtractive hybrid manufacturing technique combining 3D printed molds, casting hydrogel and sacrificial material was proposed. Compared to current fabrication techniques, the proposed method fabricated multilayered TEVG with a curved structure and multi-branches for the first time, to our best knowledge. The material of the construct was enzymatically crosslinked gelatin. The vascular geometry design was based on the model of the left coronary artery (LCA), hence the fabricated vessel-like construct possessed an interconnected channel whose diameter varied in the range of 1.8 to 3 mm. The proposed technique is a promising low-cost approach for tissue engineering.

## 2. Materials and Methods

### 2.1. Materials

Gelatin (Type A, 300 bloom from porcine skin) and Pluronic F127 was purchased from Sigma Co. Ltd. (St. Louis, MO, USA). Microbial transglutaminase (mTG) was purchased from Ajinomoto Inc. (Kanagawa, Japan); its activity was approximately 100 U/g.

Gelatin was dissolved in deionized water and was continuously stirred in a 60 °C water bath for 30 min. When the temperature of the gelatin solution dropped to 37 °C, mTG was added and thoroughly blended with the gelatin solution. The gelatin/mTG solution contains 14% (wt) gelatin and 1.4% (wt) mTG. The fugitive ink was composed of 40% (wt) Pluronic F127 in deionized water. The ink was homogenized using a mixer until the powder was fully dissolved, and then centrifuged to remove any air bubbles. The fugitive ink was subsequently loaded in a syringe and stored at 4 °C.

### 2.2. Geometry of the Tissue Engineered Vascular Grafts (TEVG)

**Figure 1:**
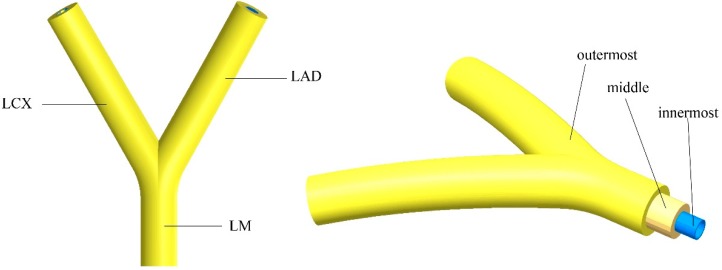
The geometry model of the proposed tissue engineered vascular grafts (TEVGs) based on left coronary artery. LCX–left circumflex; LAD–left anterior descending; LM–left main artery.

The left coronary artery (LCA) consists of the left main artery (LM) that bifurcates into the left anterior descending (LAD) and the left circumflex (LCX) [29]. The constructed geometry model is presented in Figure 1. The wall thicknesses of the middle layer and the outermost layer are set to be 0.5 mm and 0.5 mm, respectively. The innermost layer was formed by seeding human umbilical vein endothelial cells (HUVECs) on the inner wall of the channel. The dimensions of the proposed TEVG geometry are based on the clinically accurate dimensions of the LCA. The dimensions of the inner channel of the TEVG are presented in Table 1 [30]. The tapering effects were considered when constructing the LAD and LCX. The angle of 60° was used between the two branches [6]. The curvature was set to be 60 mm.

### 2.3. Fluid-Structure Interaction Simulation

**Figure 2:**
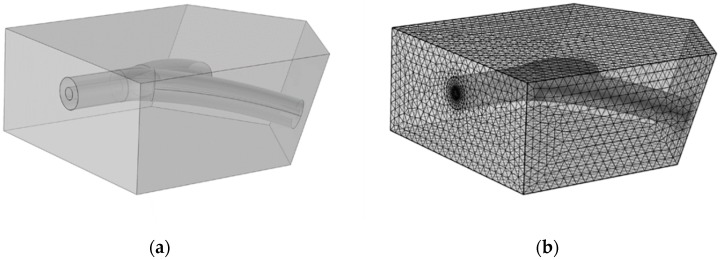
(**a**) The geometry of the bioengineered vascular construct embedded in the cardiac muscle used for the simulations, and (**b**) the mesh model created for the simulation.

The strength of a fabricated TEVG under native blood pressure is crucial for its application in the clinic. To investigate the property of the wall compliance of the constructed TEVG under the influence of the blood flow, a fluid-structure interaction (FSI) simulation was performed via COMSOL Multiphysics. The geometrical model is presented in Figure 2a along with the mesh model used in the simulation (Figure 2b). The engineered vascular construct is embedded in biological tissue, specifically the cardiac muscle. The fluid domain was also constructed. The flowing blood applies pressure to the internal surfaces, thereby deforming the vascular construct and the cardiac muscle.

The simulation consists of two distinct but coupled studies: first, a fluid-dynamics study of the blood; second, a mechanical study of the deformation of the bioengineered vascular construct and cardiac muscle. To correctly estimate the deformation response of the TEVG, the mechanical study must consider the cardiac muscle because it exerts a stiffness that resists the vascular construct deformation due to the applied pressure.

Blood was modeled as a Newtonian fluid with the use of the incompressible Navier-Stokes equations; as for LAC dimensions, the shear rate is well over the limit where blood exhibits shear-thinning behavior for the cardiac cycle. A laminar flow model was chosen as the calculated Reynolds number was below 100. The density and viscosity of the blood were set to be 1050 kg/m^3^ and 4.0 × 10^−3^ Pa∙s [31]. No slip boundary condition was imposed for the inner walls of the construct. An incompressible Neo-Hookean solid model was chosen for the TEVG and cardiac muscle so as to better predict its nonlinear stress-strain behavior. The Poisson’s ratio (υ) of the TEVG and cardiac muscle was set to be 0.45 [32,33]. As for the Lamé’s coefficients of the neo-Hookean hyperelastic materials, the Lamé’s second coefficients (μ) of the TEVG and muscle were set to be 6.20 × 10^6^ N/m^2^ and 7.20 × 10^6^ N/m^2^, respectively. Therefore, the Lamé’s first coefficients (λ) could be calculated by the following equations [34]:

(1) $$\lambda =K-\frac{2}{3}G$$

(2) $$\upsilon =\frac{3K-2G}{6K+2G}$$

(3) μ=G

where K and G represent the bulk modulus and shear modulus of the hyperelastic materials, respectively.

**Figure 3:**
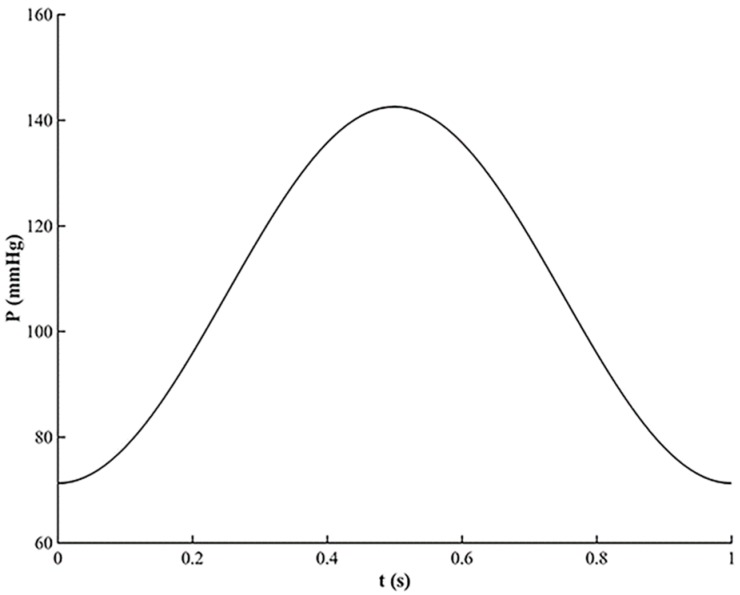
The time variation of the pressure used as the inlet boundary condition for the TEVG.

During a heart beating cycle, the pressure varies between a minimal and a maximal value [35]. Therefore, for the time-dependent analysis, a simplified waveform of the pressure for the inlet boundary condition of the fluid domain was used and is shown in Figure 3. The maximal pressure value occurs at the time t = 0.5 s. The pressure between 0 and 1 s makes the pressure vary between the minimal and maximal value during a heart beating cycle. The pressure variation during a cardiac cycle is within the range of the normotensive pulse pressure.

Additionally, the compliance of the vascular construct can be calculated as follows:

(4) $$\%\mathrm{compliance}=\frac{(R_{p_{2}}-R_{p_{1}})/R_{p_{1}}}{\left(p_{2}-p_{1}\right)}\times 10^{4}$$

where

$p_{1}$ is the lower pressure value (mmHg);
$p_{2}$ is the higher pressure value (mmHg);
$R_{p_{1}}$ is the internal radius at the lower pressure value (mm);
$R_{p_{2}}$ is the internal radius at the higher pressure value (mm).

### 2.4. Fabrication Method

#### 2.4.1. Fabrication of Mold System

**Figure 4:**
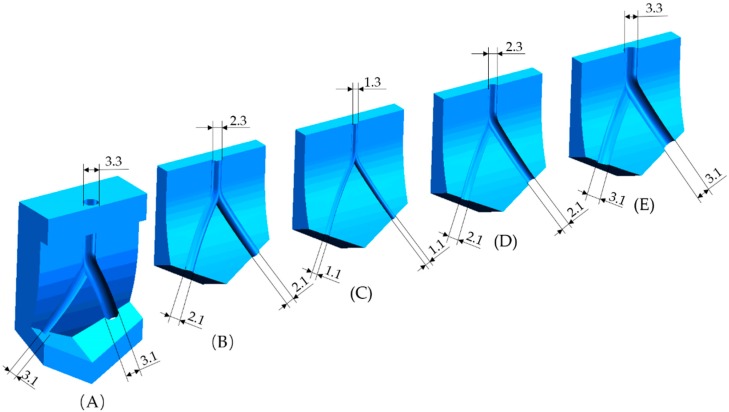
Mold system: (**A**) mold 1; (**B**) mold 2; (**C**) mold 3; (**D**) mold 4; and (**E**) mold 5.

The methods employed for the fabrication of the outermost layer and middle layer of the TEVG were realized via a mold system. The mold system consists of five separate molds. They were made of photosensitive resin (Clear Resin, Formlabs, Somerville, MA, USA) and printed directly by a 3D printer (Form 2, Formlabs) from stereolithography (STL) files (SolidWorks 2018, Dassault Systèmes, Vélizy-Villacoublay, France). The printing parameters were set following the general configurations in the program. Briefly, the layer thickness was set to be 0.025 mm and the resin temperature was 35 °C. In the settings of the supporting constructions, the density of the supports, the contact point size and the thickness of the basement were set to be 1.00, 0.7 mm and 2 mm respectively. The profiles of the five molds are shown in Figure 4. The cross section of each mold’s track is semicircular. The center trajectories of each mold’s track are identical. The curvature radiuses of the fitting surfaces of the five molds are also the same (60 mm), which corresponds to the curved structure of the bioengineered TEVG. The diameter of the groove/convex on each mold’s curved surface is determined by the dimensions of the designed TEVG.

#### 2.4.2. Fabrication of Multilayered Bifurcated TEVG with Curved Structure

**Figure 5:**
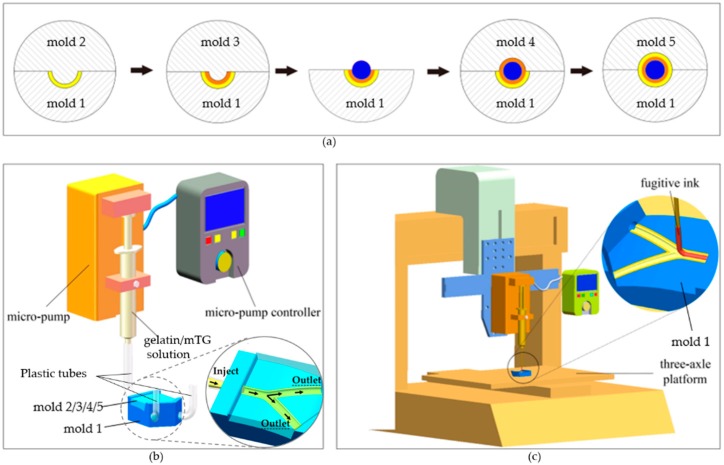
Fabrication process of the TEVG: (**a**) procedure of the step-by-step fabrication process. The molds 2–5 were gathered after mold 1 to compose the injection mold systems to form the different layers of the TEVG; (**b**) the gelatin/mTG perfusion system; (**c**) the fugitive ink printing system.

A schematic representation of the step-by-step process for the fabrication of multilayered bifurcated TEVGs with a curved structure is presented in Figure 5a. At first, mold 1 and mold 2 were fitted together, with their center trajectories coincident, and three plastic tubes were inserted into the flank hole of mold 1 as the mold side gate to compose the feed system. Next, a freshly prepared gelatin/mTG solution was housed in a syringe and pipetted into the channel formed by mold 1 and mold 2 (Figure 5b). The solution delivery rate was maintained at 6 mL/min by a micro-pump attached to the syringe. After cooling at room temperature for 15 min and then 30 min at 10 °C to induce the gelation of the gelatin injected in the molds, mold 2 was gently removed, leaving the hydrogel structure in the channel of mold 1, which corresponds to half of the outermost layer of the TEVG. In the same manner, mold 3 was fitted together with mold 1, and the gelatin/mTG solution was pipetted into the channel from the gate, gelatinating to form half of the middle layer of the structure. After the gelation of the gelatin, we removed mold 3. Then, the fugitive ink Pluronic F127 was loaded in a syringe and printed into the groove of the hydrogel on mold 1. The printing process was executed by a custom-built three-axle linkage platform (Figure 5c). The diameter of the printed F127 ink could be regulated by adjusting the moving speed of the platform and the delivery rate of the micro-pump online. After the printing process, the rest was done in the same manner as mentioned above. Briefly, mold 4 and mold 5 were subsequently fitted together with mold 1, and the gelatin/mTG solution was pipetted into the inner channel to shape the left half of the middle layer and outermost layer of the TEVG respectively. After being crosslinked at room temperature for 15 min and then at 10 °C for 30 min, the upper mold was gently removed; then, we carefully removed mold 1, and a multilayered vessel-like structure was achieved. We placed the construct at 4 °C for 20 min, and during this process, fugitive ink Pluronic F127 liquefied and flowed away, forming the inner channel of the TEVG. The obtained construct was incubated at 37 °C for 5 h to get fully crosslinked. Then, the construct was immersed in 65 °C distilled water for 5 h to heat-inactivate the residual enzyme.

### 2.5. In Vitro Cytocompatibility of the TEVG

Human umbilical cord derived endothelial cells (HUVECs) were trypsinized, centrifuged and suspended in a culture medium at a cell density of 1.0 × 10^6^ cells/mL. The cells in the third passage after defrosting were used for the experiments. The TEVG was soaked in 75% ethanol for 1 h and then washed 3 times with phosphate buffer saline (PBS) to remove the ethanol. After that, the sample was exposed to ultraviolet light for 30 min. The cell suspension was injected into the channel. After 4 h of cell attachment, the cellular construct was placed in Dulbecco’s modified Eagle medium containing high glucose and sodium pyruvate (DMEM) (HyClone, GE), supplemented with 10% fetal bovine serum (FBS) (HyClone, GE), and cultured in a humidified incubator at 37 °C. It was statically cultured with medium that was changed each day. At 4 h and 72 h after culturing, the TEVG was washed in sterile PBS (HyClone, GE) three times and stained using Live-Dye^TM^ (“live”; 1 μL/mL; BioVision) and propidium iodide (“dead”; 1 μL/mL; BioVision) for 30 min. After that, the cellular morphology and fluorescent images were observed with an inversed fluorescent microscope (Eclipse Ti-U, Nikon Instruments Inc., Tokyo, Japan).

### 2.6. Uniaxial Compressive Testing

The uniaxial compressive testing was operated on a material testing machine (Z2.5, ZWICK, Ulm, Germany). To ensure stability during the test, the diameter of the cross-section was scaled up by three times its original diameter. The uniaxial compressive tests were performed at the rate of 1 mm/min at an ambient temperature of 23 °C and 37 °C respectively. The environmental humidity was about 65%. To investigate the effect of the addition of mTG on the mechanical properties, samples with and without crosslinking were fabricated to carry out the tests.

### 2.7. Thermostability of the TEVG

To examine the thermostability of the bioengineered TEVG, samples with and without mTG were both submerged in PBS (pH 7.4) and stored in the incubator at 37 °C for different time periods (2, 4, 6, 8 and 10 days). After each time period, the samples were washed and subsequently dried in a vacuum oven at room temperature for 6 h. Their weights were recorded during this period.

## 3. Results

### 3.1. Fluid-Structure Interaction Simulation

The mechanical properties of biomimetic constructs under the effect of actual human body conditions are critical in terms of the clinical applications and potential failure. In the human body, blood vessels usually undergo very large blood pressure, especially in the case of a large-diameter vessel. Biomaterials such as gelatin usually undergo very large strains under loading, and the stress-strain relationship is generally nonlinear. The behavior of the TEVG is influenced not only by its geometry and material properties, but also by the fluid passing through it. Numerical simulations were carried out to estimate the displacement of the wall of the TEVG under the influence of the blood flow.

The inclusion of surrounding cardiac tissue is also essential for the FSI simulation process, because during the expansion process of the TEVG’s wall the cardiac muscle would exert a tethering effect on the wall and thus restrict the radial motion. Hence, the integrated simulation model is more desirable than the one consisting solely of the TEVG, thus providing more realistic boundary conditions for the simulation.

**Figure 6:**
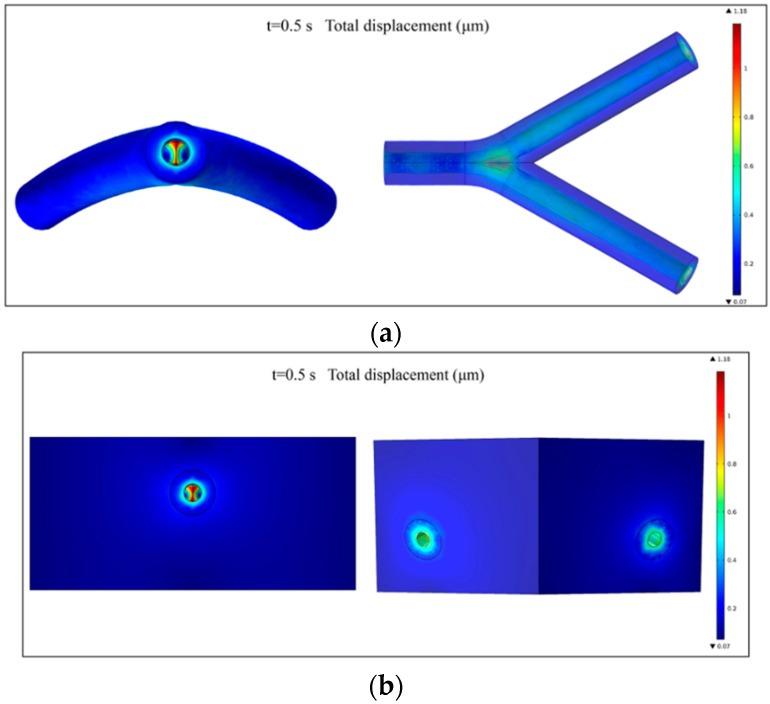
(**a**) The total displacement of the TEVG at the peak load (t = 0.5 s); and (**b**) the total displacement of the whole construct at the peak load (t = 0.5 s).

The total displacement of the TEVG and cardiac muscle at the peak pressure (t = 0.5 s) is shown in Figure 6a,b, respectively. The maximal displacement of 1.18 μm occurs at the bifurcated location. The displacement in the bifurcated area is large since this is the location where the blood flow impinges on the wall of the construct, leading to a local high-pressure region. No rupture was observed from the results, implying that the risk of failure is low under coronary blood flow conditions. The compliance of the vascular construct is calculated to be 0.26%, which is within the normal range of a human blood vessel [36].

**Figure 7:**
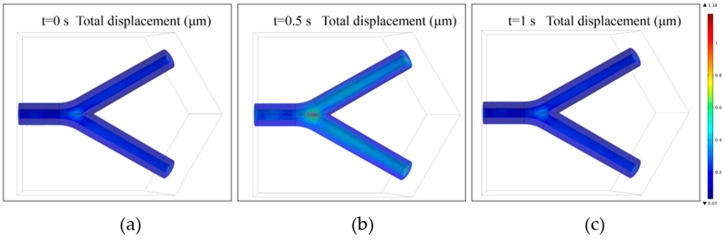
The time variation of the total displacement of the TEVG at: (**a**) t = 0 s; (**b**) t = 0.5 s; and (**c**) t = 1 s.

Additionally, for the time-dependent analysis, the total displacements of the TEVG in a complete heart beating cycle (0–1 s) are shown in Figure 7a–c. It could be observed that the peak displacement of the structure is still mainly located in the bifurcated region. The simulation results can be used to examine the risk of failure for the fabricated TEVG when it is integrated in other three-dimensional tissue constructs.

### 3.2. The Morphology of the Proposed TEVG

**Figure 8:**
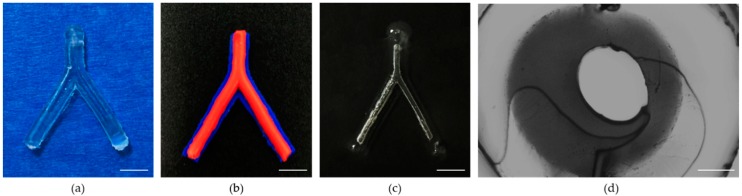
Morphology of the TEVG: (**a**) top view; (**b**) multilayered structure of the TEVG (blue: outermost layer of the structure; and red: middle layer of the structure); (**c**) inner channel of the construct after being submerged in water. (Scale bars: 5 mm); and (**d**) section view of the TEVG (Scale bar: 500 μm).

In this study, a novel method of fabricating biodegradable multilayered TEVGs was proposed. The fabricated enzymatically-crosslinked gelatin vascular construct is shown in Figure 8. The top view (Figure 8a) of the construct confirmed its bifurcated structure in the three-dimensional space. To reveal the layered structure of the construct, blue and red acrylic paints were added in the hydrogel of each layer when fabricating the construct. It can be observed that the TEVG possesses a distinct multilayered structure (Figure 8b, blue: outermost layer of the structure; and red: middle layer of the structure). The inner channel of the construct became visible after being immersed in water (Figure 8c), which demonstrated its connectivity. The section view of the TEVG revealed that the molding generated a real circular shape after sacrificing the F-127 (Figure 8d) and form a bifurcated channel ready for liquid perfusion (Video S1). The innermost layer partially deviated from the center, which was supposed to be affected by the die clearance. Compared with previously seen TEVGs, the proposed TEVGs possess a branched structure while keeping the multilayered feature. This is of particular importance because over-simplified in vitro TEVG models have a limited significance for simulating the actual in vivo environment.

### 3.3. The Results after Cells Seeding

**Figure 9:**
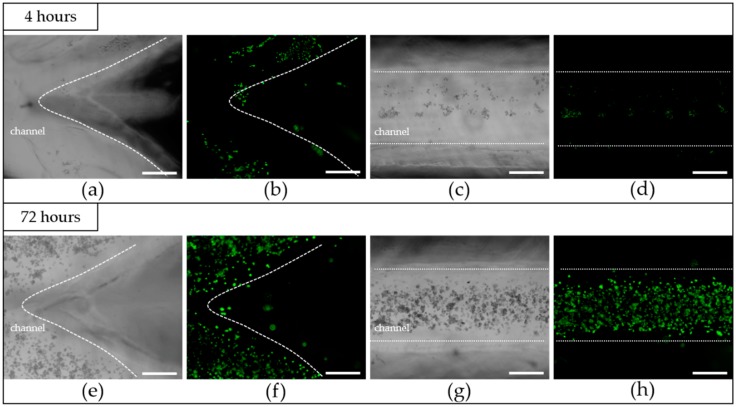
Microscopic images and fluorescent images of cells; the dotted lines were used to divide the inside and outside of the channel: (**a**,**b**) after 4 h of attachment at the bifurcate area of the TEVG, (**c**,**d**) after 4 h of attachment at the linear channel of the TEVG, (**e**,**f**) after 72 h of culturing at the bifurcate area of the TEVG, (**g**,**h**) after 72 h of culturing at the linear channel of the TEVG. (Scale bars: 500 μm).

One of the most critical properties of biomimetic structures are their in vitro cytocompatibility, and the creation of a continuous monolayer of ECs in the inner channel is necessary for the proper function of TEVGs [37]. To investigate the in vitro cytocompatibility of the fabricated TEVGs, HUVECs were seeded and statically cultured in the inner channel. The microscopic morphology image (Figure 9a,c) and the fluorescent image (Figure 9b,d) of endothelial cells after 4 h of attachment were shown. It could be observed that the HUVECs adhere well on the lumina. However, the cell distribution at this stage is quite nonuniform, and this might be caused by the uneven distribution of the cell in the suspension at the initial stage. Additionally, because the inner diameter of the channel is quite large, the adhesion of the cells is difficult. Many cells entering from the inlet didn’t have the chance to adhere and were flushed away from the outlet. After 72 h of culturing, as could be observed from Figure 9e–h, the amount of HUVECs had significantly increased, and the cells in the inner channel were found to have well spread on the wall and to have taken a normal cellular phenotype. In spite of the nonuniformity of the distribution in the attachment phase, the cells still covered the surface of the channel and formed an endothelialized monolayer. The results indicated that the crosslinked TEVG had a good in vitro cytocompatibility and was suitable for cell attachment and for proliferation.

### 3.4. Mechanical Properties of the Proposed TEVG

**Figure 10:**
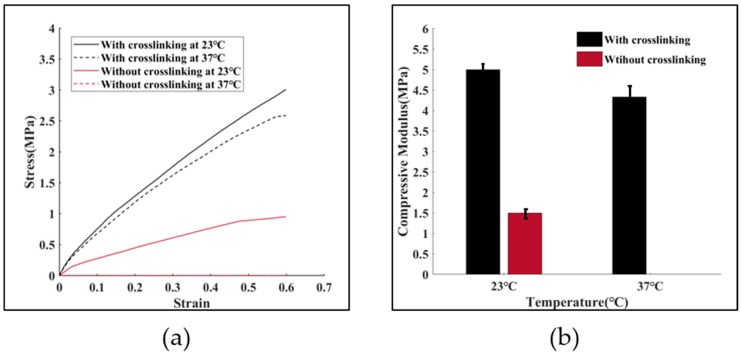
Compressive testing. (**a**) stress-strain curves of the samples with or without crosslinking at 23 °C and 37 °C respectively; and (**b**) compressive modulus of the samples with or without crosslinking at 23 °C and 37 °C respectively.

The mechanical properties of tissue-engineered biological constructs determine their application fields. The TEVGs fabricated in this study possess a high aspect ratio, which determines their susceptibility to bucking when suffering from a compressive load. Therefore, the uniaxial compressive mechanical properties of the fabricated TEVG under room temperature and physiological conditions were investigated. The mechanical properties of gelatin samples with and without crosslinking were evaluated. Figure 10a shows the compressive stress-strain curves for up to a 60% strain. The compressive modulus of the four groups of samples are shown in Figure 10b. It can be observed that the addition of mTG (1.4%, wt) increased the compressive modulus of the constructs by more than three times, from 1.5 MPa to 5 MPa at room temperature. Under 37 °C, the mechanical properties of the gelatin/mTG TEVG is almost the same as that under 23 °C, while the pure gelatin one completely solved. The main reason for this mechanical enhancement lies in the fact that the composition of gelatin includes glutamine and lysine. The presence of these amino acids means there are more crosslinking sites in gelatin, thus promoting the chance of creating stiffer gels when provided with sufficient amounts of mTG under proper crosslinking conditions. Compared with the TEVG composed of GelMA hydrogels, the maximum compressive modulus of the gelatin/mTG based TEVG is enhanced more than ten-fold [38].

The mechanical properties of the enzymatically-crosslinked TEVG are still relatively low compared to those of native blood vessels. However, as was envisioned, the real value of the fabricated vessel-mimic structure lies in its capacity to carry various types of vascular cells and to eventually develop into function blood vessels. Hence, the capability of maintaining its original morphology when integrated with other engineered scaffolds is sufficient for the proper function of the fabricated TEVG.

### 3.5. Thermostability

**Figure 11:**
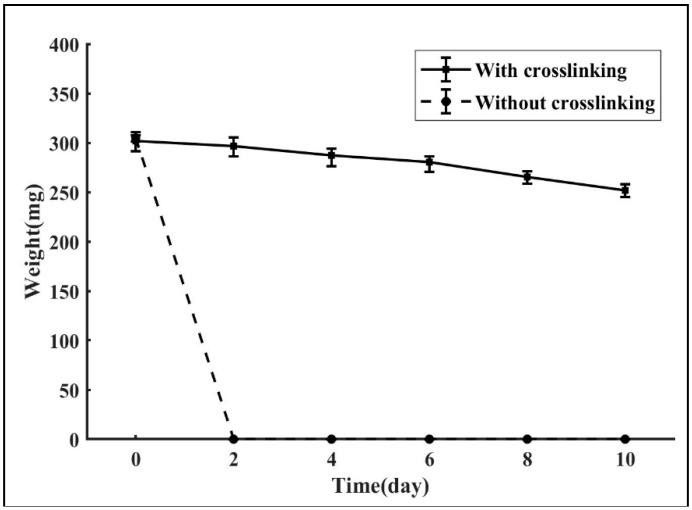
In vitro degradation of the TEVG with and without crosslinking.

One possible application of the fabricated TEVG is its potential of being integrated into other three-dimensional scaffolds. As the carrier of different types of vascular cells, the designed TEVG has the potential of developing into a functional multilayered blood vessel. One crucial premise is the ability of maintaining its design morphology under the temperature of the human body. Figure 11 shows the in vitro degradation process of the gelatin samples with and without crosslinking. It could be observed that the enzymatically-crosslinked TEVGs degrade much slower than those without crosslinking, with a degradation rate of 16.54%. In contrast, the samples composed purely of gelatin completely dissolved within two days. One possible explanation for the enhancement is that the addition of mTG increased the intermolecular association through the formation of covalent bonds in the gel matrix, thus dramatically slowing down the degradation speed of the gelatin construction. The results indicate that the gelatin/mTG biopolymer possesses a distinct advantage over the pure gelatin polymer in terms of thermostability, which proved the feasibility of using the crosslinked construct as the carrier of cells to form a functional vessel.

## 4. Discussion

The biological compositions of each layer of the native vessels are different. Likewise, the wall thicknesses and mechanical properties of different layers also vary. For instance, the wall thickness of the vessels near the heart are relatively larger. Consequently, when fabricating TEVGs, the ability to independently control each layer’s composition and thickness is essential in order to achieve their biological and mechanical performances such as the biocompatibility and the compressive strength.

Today, extrusion-based printing [39], inkjet printing [40], stereolithography [41], two-photon polymerization [42] and laser-assisted printing [43] have been used wisely in vasculature engineering. While the above methods successfully shape tissue-engineered vessels having a multilayered structure or achieve vascularization in bulk constructions, there remains the challenge of fabricating curved bifurcated vascular constructions with a multilayered wall at the macro-scale in a controllable way. In the current study, a novel method of fabricating multilayered biodegradable TEVGs with a curved structure and multi-branches was proposed. The mold system was designed by computer aided design (CAD) software and fabricated by a 3D printer. Taking advantage of the merits of 3D printing technology, we can create three-dimensional structures that are closer to the native blood vessel’s morphologies. The proposed method also allows the variation of the composition and dimension of each layer of the constructs. By varying the design parameters of each layer, for example, or by possibly incorporating various bioactive substances in each layer, the thickness and biocompatibility of each layer can be independently controlled. Furthermore, the fabricated tissue-engineered TEVGs possessed a bifurcated structure, which was determined by the CAD models of the mold system. Thus, it is also possible to fabricate vascular constructs with more branches by simply modifying the CAD models. In addition, the three-dimensional structure of the constructs could also be easily modified by varying the design parameters of the mold system. Because of this design flexibility, 3D printed mold systems will give researchers the ability to quickly and inexpensively design TEVGs with the shape and complexity they desire.

In recent years, several methodologies have been introduced to create channels in bulk hydrogels. Bertassoni et al. embedded agarose stripes in in the hydrogel construction to form the inner networks [44]. Negrini et al. sacrificed alginate templates through a chelating agent to obtain a porous gelatin hydrogel [45]. Li et al. added a pre-printed bifurcated polyvinyl alcohol (PVA) structure to the hydrogel to realize vascularization [46]. Compared with the fugitive materials listed above, the adoption of the Pluronic F127 fugitive ink as the sacrificial material is an easy way of forming the inner channel, as the Pluronic F127 is a thermal sensitive material. Furthermore, by taking advantage of the strong shear thinning response of F127 at room temperature, F127 can be printed into very complex channel networks and maintains its shape fidelity without harmful chemical cross-linking, heat processing or additional templates preparing to fabricate inner networks. For TEVGs below the LCST, the ink liquefies and flows readily, while at the same time the other hydrogel materials used are stiff and solid-like, which makes it easily removed by temperature variation without being obstructed by the closed structure and without exerting extra mechanical force that may impair the printed structure. Taking advantage of this complimentary behavior, the printed ink could be removed without influencing the form of other materials. Our work successfully solved the problem of creating multiple branches for a multi-layered TEVG, which means that the fabricated TEVGs possess more features similar to the native blood vessel. This is a big step towards creating TEVGs with the same level of complexity, which has a tremendous significance for in vitro cardiovascular research and could hopefully reduce the demand for animal experiments.

HUVECs were found to adhere well to the luminal surface of the sample. The formed continuous monolayer of HUVECs was evident after 72 h, which demonstrated the good biocompatibility of the enzymatically-crosslinked TEVGs and their potential for acting as a substitute for ECM.

From the acquired results, one can figure out that the proposed triple-layered TEVG could easily be imbedded into other porous tissue-engineered scaffolds, thus forming a multi-scale vasculature within a whole three-dimensional structure; the scale of the whole vasculature could range from micrometers to millimeters. The existence of this multi-scale vasculature will greatly facilitate the mass transport within the scaffold. Furthermore, when combined with techniques such as cell encapsulation, the fabricated construct would have the potential of developing into a real functional blood vessel. Specifically, if the cell-encapsulating hydrogels were used for the fabrication of the TEVG, different types of vascular cells would further develop into different layers of the blood vessel with the degradation of the hydrogel materials.

As for the scale of the proposed construct, whether the smallest inner diameter could be achieved by this technique depends to a certain degree on the resolution of the 3D printing technology. Theoretically speaking, by promoting the accuracy of the 3D printer, or by adopting techniques with a higher precision such as micro-fluids technology, the scale range of the inner diameter of the TEVG could be further expanded, thus widening the application field of this novel process. Hence, this fabrication technique holds great potential for impacting a wide range of fields.

Finally, another promising advantage of this novel technique lies in its capacity to design patient specific vessel models because the mold can be constructed by a 3D printer which could be driven directly by the computed tomography (CT) scan data of patients’ original vessels, which means that personalized TEVGs that are more suited to the actual human body conditions can be constructed. Constructing the mold system from the clinical data of native vessels is an interesting point for future exploration, and this personalized diagnostic approach has tremendous potential in clinical diagnoses and treatments.

## 5. Conclusions

In this study, a novel approach for fabricating a multilayered biodegradable bioengineered vascular construct with a cured structure and multi-branches was presented. The method used in this study combined 3D printed molds, casting hydrogel and sacrificial material, providing an easy technique to construct TEVGs whose morphology and structure are close to those of the original blood vessels. With the inherent advantages of 3D printing, it is envisioned that the proposed technique will play a significant role in the field of tissue engineering and in assisting studies on cardiovascular diseases.

## Figures and Tables

**Table 1:** Dimensions of the inner channel of the tissue engineered vascular grafts (TEVGs).

Vessel	Diameter (mm)		Length (mm)	Curvature Radius (mm)
	Inlet	Outlet		
Left main artery (LM)	1.3	1.3	6	60
Left circumflex (LCX)	1.3	1.1	15	60
Left anterior descending (LAD)	1.3	1.1	15	60

## Supplementary Materials

The following are available online at https://www.mdpi.com/2072-666X/10/4/275/s1, Video S1: Liquid perfusion inside the channels.
